# Largest Documented Peri-Aortic Hematoma Following the Bentall Procedure: A Case Report and Review of Surveillance Strategies

**DOI:** 10.7759/cureus.82273

**Published:** 2025-04-14

**Authors:** Emilio Abi Rached, Avtandil Kochiashvili, Mohamed Jailani, Mohiuddin Cheema

**Affiliations:** 1 Internal Medicine, University of Connecticut, Farmington, USA; 2 Cardiac Surgery, Hartford Hospital, Hartford, USA

**Keywords:** anastomotic leak, cardiac surgery complications, imaging surveillance, peri-aortic hematoma, pseudo-aneurysm, thoracic aortic aneurysm repair

## Abstract

Anastomotic graft leaks following thoracic aortic repair are rare but potentially life-threatening complications that may present with subtle symptoms, leading to delayed diagnosis. We present the case of a 74-year-old male who developed progressive dyspnea eight months after undergoing a Bentall procedure for an ascending aortic aneurysm. Imaging revealed a massive 13.2 × 11.2 × 11.5 cm contained peri-aortic hematoma, causing significant mass effect on nearby structures, which was successfully surgically repaired. This case represents the largest reported peri-aortic hematoma following Bentall surgery and highlights the challenges in detecting and managing late complications of open thoracic aortic repair, given the lack of standardized surveillance protocols for anastomotic open graft leaks.

## Introduction

Anastomotic graft leaks following thoracic aortic repair or replacement are uncommon but potentially life-threatening complications, occurring in up to 4.3% of cases [1]. The severity of these leaks depends on factors such as the rate of expansion, size, location, and their capacity to compress adjacent structures. While endoleaks, defined as the persistence of blood flow outside the lumen of an endovascular stent graft within the aneurysm sac, are well-documented complications of thoracic endovascular aortic repair (TEVAR), large graft leaks following open surgical repair are less frequently reported, likely due to their subtle clinical presentation, which makes diagnosis more challenging. As a result, they may be underrecognized in clinical practice.

The Bentall procedure, first described by Hugh Bentall in 1968, is a composite graft replacement technique used to treat pathology of the aortic root, typically aneurysmal disease or dissection involving the aortic valve, root, and ascending aorta. The procedure involves replacing the diseased aortic root and valve with a composite graft, along with reimplantation of the coronary arteries into the graft [2,3]. Aneurysm graft leaks can present with subtle and nonspecific symptoms, such as mild dyspnea, making early diagnosis challenging. This can lead to significant complications, such as pseudoaneurysm formation or mass effect on nearby structures [2,3]. Despite the clinical significance of anastomotic leaks, guidelines for the optimal timing and modality of post-surgical surveillance imaging following open thoracic aortic repair, like the 2022 ACC/AHA (American College of Cardiology/American Heart Association) and 2023 ESC (European Society of Cardiology) guidelines for management of aortic diseases, remain inconsistent, whereas in TEVAR, lifelong annual surveillance with CTA (computed tomography angiography) or MRA (magnetic resonance angiography) is recommended [4,5].

In this report, we present the largest documented contained hematoma following a Bentall procedure, highlighting the potential for insidious clinical progression in post-surgical aortic patients. Our goal is to aid providers in recognizing chronic mass effect symptoms and in considering late anastomotic leaks in the differential diagnosis of patients with prior thoracic aortic surgery presenting with unexplained dyspnea.

## Case presentation

A 74-year-old male with a history of hypertension, gout, paroxysmal atrial fibrillation (on apixaban), and an elective thoracic ascending aortic aneurysm repair by the Bentall procedure eight months prior, presented to the emergency department with a one-week history of mild shortness of breath on exertion. Previously, he was in his usual state of health, able to perform activities of daily life with no restrictions. There was no associated cough, sputum production, rhinorrhea, fever, chest pain, palpitations, orthopnea, lower limb edema, or facial or upper extremity swelling. His vital signs showed a blood pressure of 164/89 mmHg, a heart rate of 68 bpm, a temperature of 97.4°F, and an oxygen saturation of 95% on room air. A comprehensive physical examination identified decreased air entry in the right lower lobe on lung auscultation. No other abnormalities were found.

Laboratory work-up showed an elevated white blood cell count, chronic stable normocytic anemia with baseline hemoglobin of 9 g/dL, normal electrolytes, creatinine, liver function tests, and troponin T along with an elevated NT-proBNP (Table 1). 

**Table 1:** Laboratory results of the patient on admission AST: aspartate transaminase, ALT: alanine transaminase, ALP: alkaline phosphatase, NT-proBNP: N-terminal pro-B-type natriuretic peptide, INR: international normalized ratio.

Blood analysis	Patient’s results	Normal range
White blood cells (× 10^9^/L)	7.8	4–11
Hemoglobin (g/dL)	8.9	13.0–17.7
Hematocrit (%)	29.4	39–54
Platelets (× 10^9^/L)	445	150–450
MCV (fL)	85	80–100
Creatinine (mg/dL)	0.8	0.5–1.3
AST (U/L)	26	10–55
ALT (U/L)	28	10–55
ALP (U/L)	126	45–128
Total bilirubin (mg/dL)	0.5	0.2–1.0
High-sensitivity troponin T (ng/dL)	19	<23
NT-proBNP (pg/mL)	1,027	<125
INR	1.6	INR therapeutic ranges: standard dose anticoagulant 2.0 to 3.0

**Figure 1:**
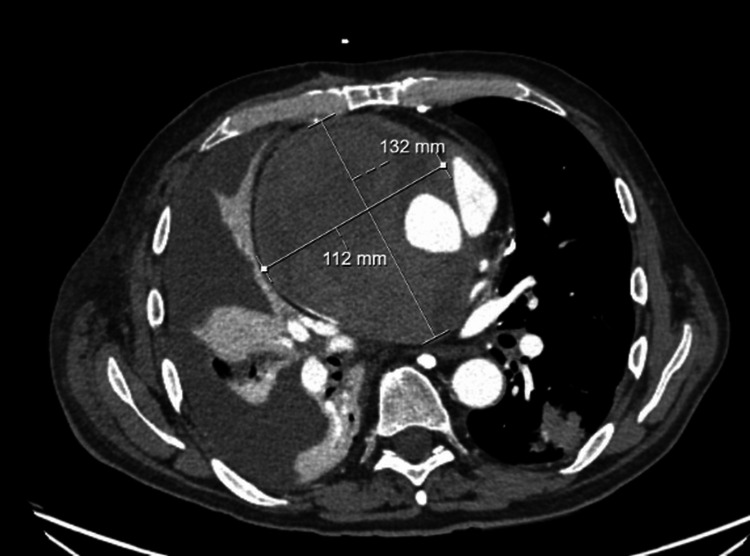
CT aorta on presentation A 13.2 × 11.2 × 11.5 cm graft leak into a partially thrombosed pseudoaneurysm/hematoma of the ascending aorta, with compression of the right upper lobe, right pulmonary artery, proximal right coronary artery, and superior vena cava.

His electrocardiogram showed normal sinus rhythm with no T-wave abnormality. Initial computed tomography (CT) angiography of the chest showed a large, dense collection around the ascending aorta graft, likely related to a graft leak, accompanied by a large right-sided pleural effusion. An aortic CT was performed and confirmed a large 13.2 × 11.2 × 11.5 cm graft leak into a partially thrombosed hematoma of the ascending aorta, causing significant mass effect with compression of the right upper lobe, right pulmonary artery, proximal right coronary artery, and almost complete collapse of the superior vena cava (Figure 1). The delayed venous phase suggested a relatively slow leak around the repaired ascending aorta. A diagnostic and therapeutic right-sided thoracentesis was performed and yielded 1.1 L of amber-colored pleural fluid with 8,000 red blood cells, described as a non-hemorrhagic, blood-tinged effusion. A transthoracic echocardiogram showed evident flow entering the pseudoaneurysm superior to the right coronary cusp, elevated right ventricular (RV) flow due to compression of the RV outflow tract and right pulmonary artery, a decreased tricuspid annular plane systolic excursion (TAPSE) of 14 mm, and a low-normal ejection fraction of 50-54%, with a normal gradient across the prosthetic aortic valve.

**Figure 2:**
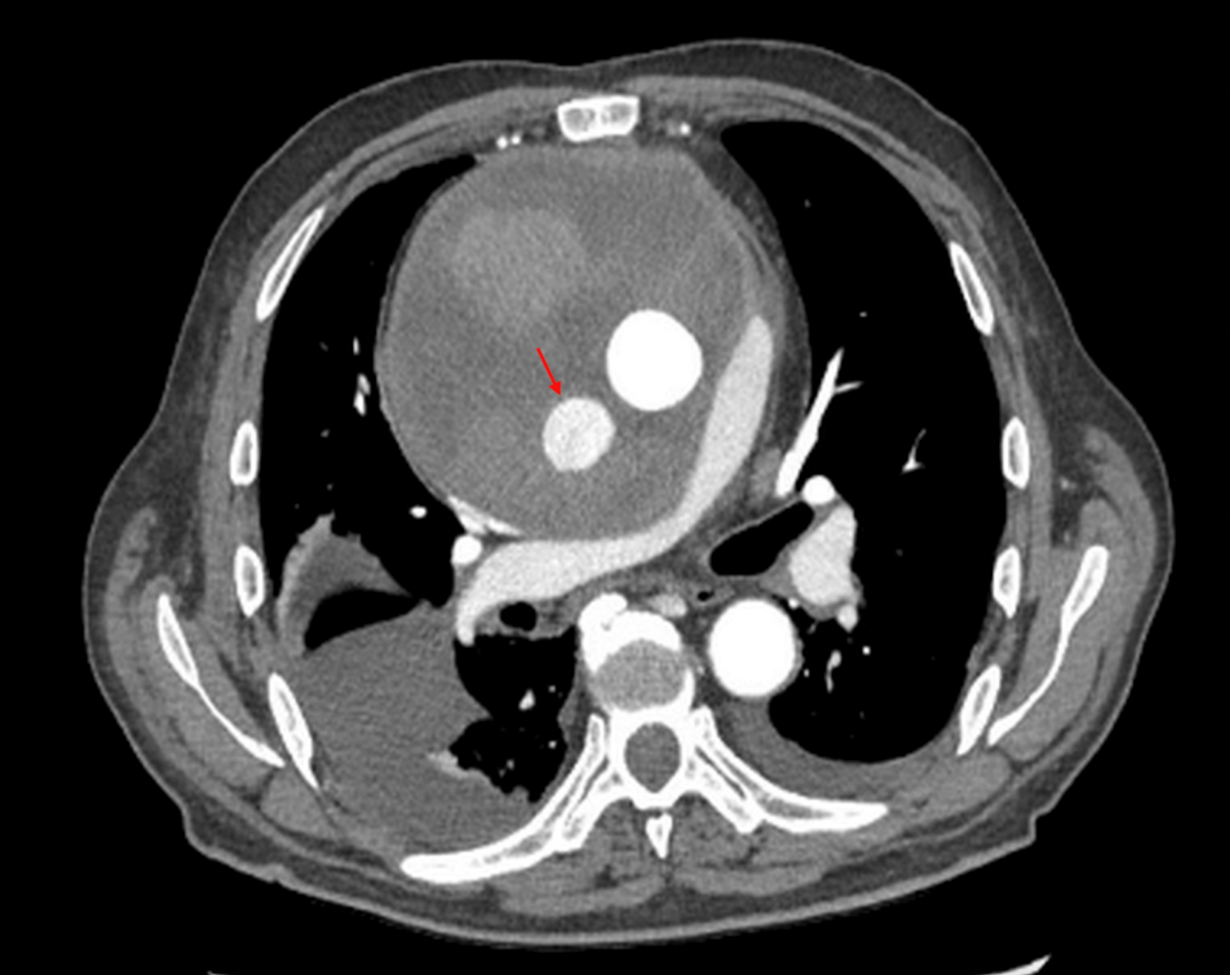
Repeat CT aorta three days after presentation A 3 cm enhancing pseudoaneurysm arising from the posterolateral aspect of the ascending aorta (red arrow), with an increase in the size of the hematoma by 0.5 cm in each dimension.

**Figure 3:**
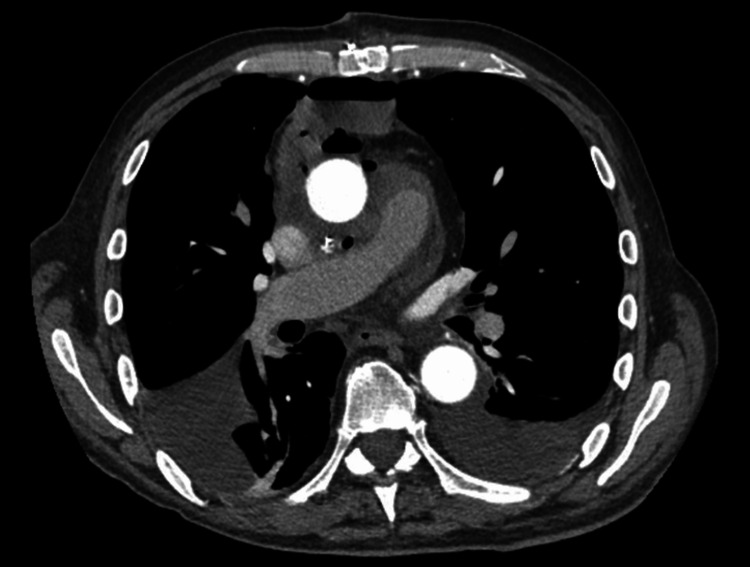
CT aorta 10 days after repair Normal-caliber thoracic aorta with no evidence of extravasation and a significant decrease in the size of the hematoma.

The cardiothoracic surgery team was consulted, and given the stable, contained, and likely chronic nature of the leak, the decision was made to admit the patient for close observation. Anticoagulation was held, and reversal agents such as prothrombin complex concentrate (PCC) were not administered, given the patient’s hemodynamic stability. His vital signs, physical examination, and laboratory work-up remained stable. A follow-up aortic CT performed three days later revealed a new 3 cm enhancing pseudoaneurysm arising from the posterolateral aspect of the ascending aorta, positioned within the known periaortic hematoma, representing a fresh bleed with an increase in the size of the hematoma by 0.5 cm in each dimension (Figure 2). Subsequently, the patient underwent a redo sternotomy for evacuation of the hematoma and repair of the ascending pseudoaneurysm. Intraoperatively, the mediastinal hematoma was opened, and an old organized thrombus as well as a fresh thrombus were evacuated. Active extravasation from the distal aortic graft suture line to the arch posteriorly was seen, along with a small pulsatile bleed from the right coronary button. Both were repaired with interrupted pledgeted sutures, with no residual bleeding noted. The graft material was intact, with no signs of dehiscence at the suture sites. The procedure was complicated by a hemodynamically stable pericardial hematoma that was surgically evacuated. The patient was weaned off inotropic support and extubated on postoperative day one. A repeat CT angiography of the chest 10 days later showed a thoracic aorta normal in caliber with no observed leaks or extravasation, along with a significant decrease in the size of the hematoma (Figure 3). The patient's hemoglobin stabilized around his baseline at the time of the repeat CT scan without the need for transfusions. The patient was able to ambulate independently with improvement in his shortness of breath and was discharged home with close cardiology and cardiothoracic outpatient follow-up. He was scheduled to resume apixaban four weeks after discharge, with a follow-up aortic CT scan planned in three months.

## Discussion

The Bentall procedure is a well-established surgical technique for the management of aortic root pathologies, including aortic root aneurysms and aortic valve disease. It involves composite graft replacement of the aortic root and valve, along with coronary artery reimplantation [6]. Its main indications include ascending aortic aneurysms, aortic dissections involving the root, and conditions such as Marfan syndrome or bicuspid aortic valve disease with root dilation [6,7]. It carries a risk of several complications, some of which can be life-threatening. According to a systematic review and meta-analysis by Mookhoek et al., the overall early mortality rate after the Bentall procedure is approximately 5.8%, with rates varying based on patient comorbidities and surgical complexity [6]. Major complications include stroke (3.5-5.0%), myocardial infarction (2.4-4.8%), renal failure requiring dialysis (4.3%), and reoperations for bleeding (6.2%) [6]. Peri-graft hematomas and pseudoaneurysms due to graft leaks are less common and may occur in 2-4% of cases, but can pose significant long-term risks such as progressive aneurysmal expansion, compression of adjacent structures, graft infection, or even delayed rupture [6,8].

Peri-graft hematomas are defined as collections of extravasated blood around the aortic graft, typically resulting from minor anastomotic leaks [9]. While many leaks remain clinically silent, they can progress to pseudoaneurysm formation in 2-4% of cases [2,3]. Common leakage sites after the Bentall procedure include the coronary artery reimplantation sites and the suture line between the native aorta and the synthetic graft [2,3]. These leaks differ from endovascular repair (TEVAR) leaks, which are categorized into five types: type I (inadequate seal at graft attachment), type II (retrograde flow from branch vessels), type III (graft fabric defects), type IV (graft porosity), and type V (endotension) [4,10]. Unlike TEVAR-related endoleaks, leaks following open surgical repair often remain contained within mediastinal adhesions, leading to chronic, expanding pseudoaneurysms that are often discovered incidentally on imaging or present with complications from compression of adjacent structures, as seen in our case [2]. Anastomotic leaks following the Bentall procedure typically develop within the first few weeks to months after surgery. Small, stable hematomas identified within the first 1-2 weeks are common and often resolve spontaneously, whereas new or enlarging hematomas detected beyond 6-8 weeks are concerning for ongoing graft leak and usually warrant further evaluation or intervention, as in our case [2,6,8].

To date, the largest reported giant ascending aortic aneurysm in the literature measured 14 cm in diameter [1]. However, data on the size of peri-aortic hematomas and pseudoaneurysm formation following surgical thoracic aortic repair remain scarce, with the largest reported hematoma after a Bentall procedure measuring 10 × 8.5 × 4.7 cm in an 11-year-old patient with Marfan syndrome [7]. To our knowledge, our case represents the largest ever reported aneurysm graft leak following the Bentall procedure. In our patient, chronic hypertension and the use of anticoagulation may have contributed to both the development of the graft leak and the extent of the associated hematoma. Elevated systemic pressures could increase mechanical stress along the graft suture lines, while anticoagulation may exacerbate bleeding from even a minor leak.

Surveillance imaging after surgical thoracic aortic repair, including the Bentall procedure, is essential for detecting late complications such as graft leak and pseudoaneurysm. However, guidelines for the optimal timing and modality remain inconsistent [3]. Different schemes have been proposed, with some institutions recommending initial CT angiography within the first year postoperatively, followed by interval imaging every 3-5 years unless new symptoms develop [3,11]. In contrast, TEVAR patients are recommended to undergo routine post-procedural imaging at 1 month, 6 months, and 12 months, followed by lifelong annual surveillance with CTA or MRA due to the higher risk of delayed complications such as endoleaks, stent migration, and graft collapse [4,5]. The 2023 ESC guidelines advocate for a risk-stratified approach, with extended surveillance particularly in patients with connective tissue disease or complex aortic pathology, whereas the 2022 AHA guidelines suggest a more structured surveillance plan with imaging at 1 month, 6 months, and annually after thoracic aortic repair [6,9].

Given the unpredictable timing of late complications following open thoracic aortic repair, along with the subtle symptoms they can present with, our case highlights a possible role for a more structured and possibly prolonged imaging surveillance strategy in these patients. An approach incorporating both routine periodic imaging and individualized risk-based monitoring may improve early detection of complications such as peri-graft hematomas and pseudoaneurysms, potentially preventing catastrophic outcomes, including the need for high-risk reoperation, which carries a reported mortality of up to 8%. A proposed strategy could include routine CT angiography or MR angiography at 3 months, 6 months, 12 months, and annually thereafter, with consideration for extending surveillance beyond 5 years, particularly in patients with connective tissue disease, complex repairs, or prior graft-related complications. Standardized imaging could help identify clinically significant sequelae of peri-graft collections, such as superior vena cava syndrome, airway compression, cardiac tamponade, or progressive aneurysmal expansion, allowing for timely intervention.

## Conclusions

Large, contained thoracic aorta graft leaks are uncommon complications that can chronically expand and cause significant compression of intrathoracic vasculature, heart, and lungs. With no standardized guidelines for long-term imaging surveillance after open surgical repair, there is a risk of delayed diagnosis due to the often subtle and insidious clinical presentation of such leaks. Our report, documenting the largest known peri-graft hematoma following Bentall surgery, emphasizes the possible role of more robust strategies for postoperative surveillance. Implementing a standardized imaging protocol that combines routine follow-up with personalized risk assessment could facilitate earlier detection of complications, ultimately improving patient outcomes and reducing the risks of reoperation and mortality associated with these rare but serious complications.
